# Discrete Element Simulation of Fracture Evolution around a Borehole for Gas Extraction in Coal Seams

**DOI:** 10.1021/acsomega.2c03623

**Published:** 2022-10-18

**Authors:** Zehua Wang, Hongqing Cui, Guoying Wei, Tianrang Jia, Luyao Wang

**Affiliations:** † School of Safety Science and Engineering, Henan Polytechnic University, Jiaozuo 454000, Henan, China; ‡ State Key Laboratory Cultivation Base for Gas Geology and Gas Control, Henan Polytechnic University, Jiaozuo 454000, Henan, China; § Central Plains Economic Region Coalbed (Shale) Gas Innovation Center, Jiaozuo 454000, Henan, China; ∥ JiaoZuo College Of Industry And Trade, Jiaozuo 454000, Henan, China

## Abstract

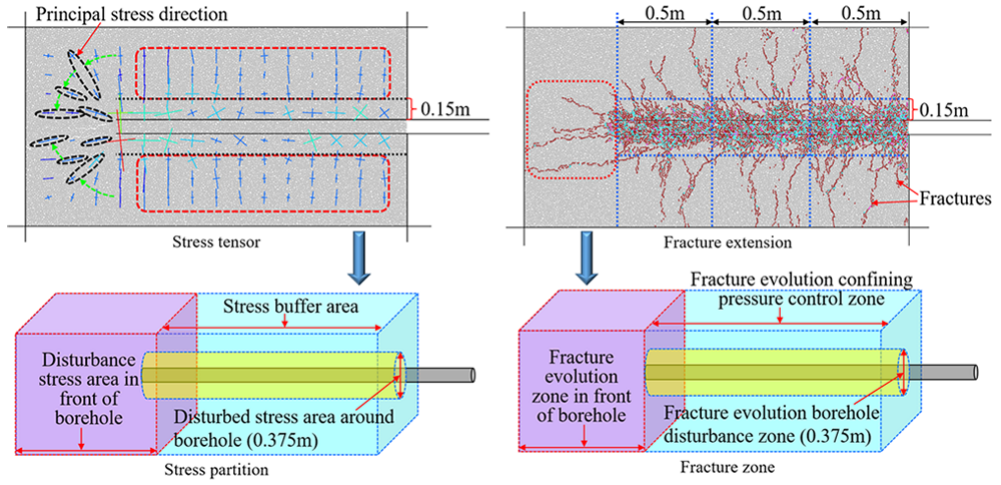

The stability analysis of underground caverns, tunnels, and boreholes is of great significance to underground engineering. In order to study the stress change of the coal around a hole and the evolution law of fractures during the coal seam drilling process, the discrete element simulation of the coal seam drilling process was carried out by using the particle flow code (PFC^2D^). The results show that during the drilling process, under the influence of confining pressure and drilling disturbance, the coal stress field around the hole and the development of fractures around the hole have the characteristics of zoning and dynamic evolution. In the axial direction of the borehole, it is divided into front and rear areas, and in the vertical axial direction, it is divided into the drilling disturbance zone and the confining pressure main control zone. During the drilling process, the direction of the maximum principal stress in the front zone gradually changes from the vertical hole axis to the direction parallel to the hole axis, and tension fractures are mainly developed along the drilling direction. In the rear zone, the principal stress direction tends to be stable and the principal stress value undergoes dynamic changes, and a large number of vertical hole axis tension fractures are developed. The drilling disturbance zone appears near the hole wall and has an important influence on the stability of the hole wall, while the confining pressure main control zone determines the antireflection effect around the hole and the influence radius of the hole. This work helps the understanding of the damage range and failure characteristics of the surrounding rock during the drilling process and has great significance for the guidance of drilling design.

## 1 Introduction

**Figure 1:**
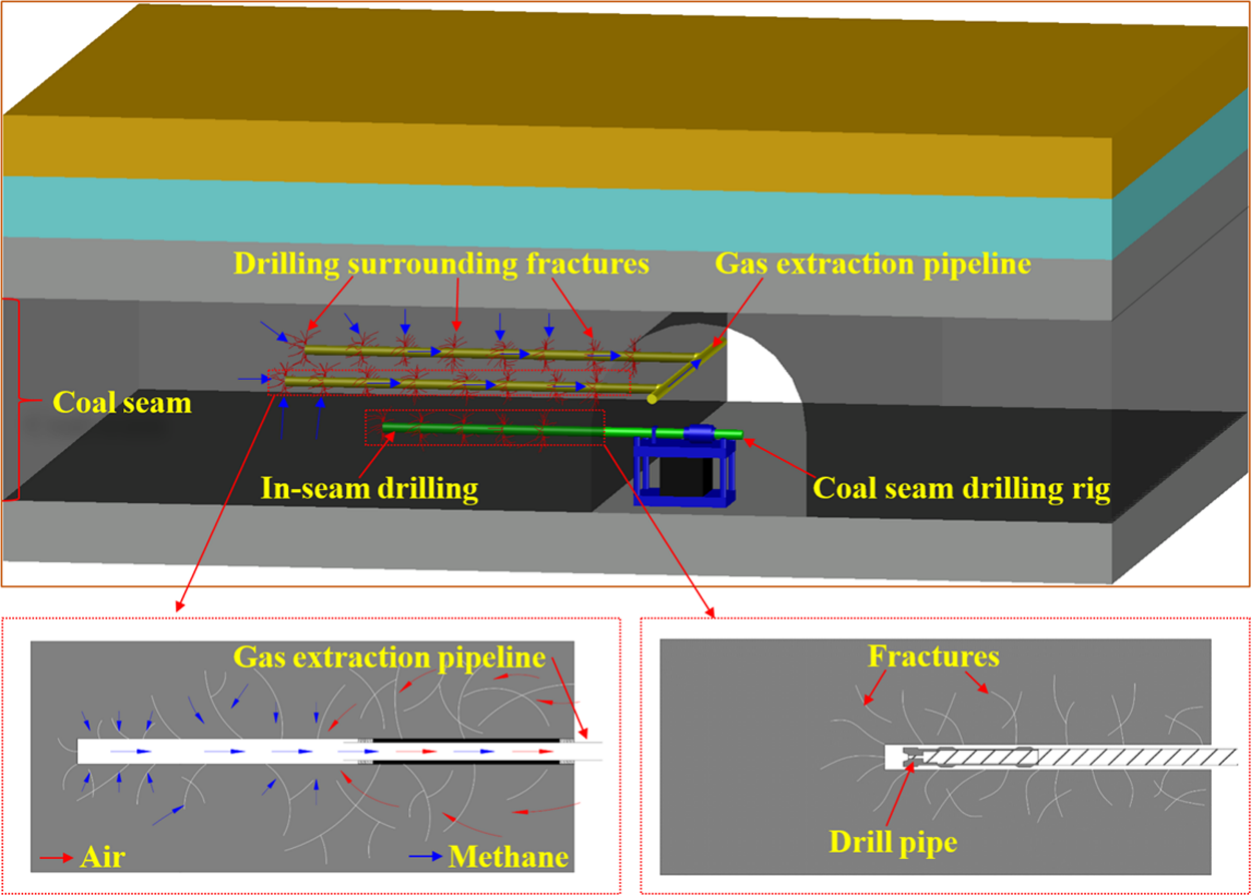
Schematic diagram of a drilling and gas drainage system in a coal seam.

Coal seam gas is an important factor that causes disasters in coal mines but is also an important unconventional natural gas resource.^1−3^ Gas extraction in coal seams is the most effective way to prevent and control gas disasters and also the most important means to exploit coal seam gas.^4,5^ The construction of gas extraction boreholes in coal seams changes the stress state of the surrounding rock of boreholes, leads to the destruction of coal and the expansion and development of fractures in coal, and forms a complex fracture system. The development of fracture systems directly affects the gas extraction efficiency of coal seams (Figure 1).

In order to study the stress state and failure mode around a hole, many researchers have done much theoretical and experimental research on the rock failure around the hole.^6−8^ In addition, experiments under uniaxial and biaxial compressive loading conditions also have been carried out to study the fracture evolution around the hole.^9−12^ The results of these experiments show that not only tensile fractures but also shear fractures exist around boreholes. The shape and size of the borehole and the stress state have a great influence on the mechanical properties of the rock sample. Li et al.^13^ have systematically studied the influence of several key factors, such as borehole diameter, far-field stress, and rock heterogeneity, on borehole failure. Lu et al.^14^ and Yang et al.^15^ have studied the mechanical properties of specimens with holes under true triaxial loading and the failure behavior and fracture evolution mechanism of nonsustained jointed rock masses with round holes, respectively. Yin et al.^16^ studied the uniaxial compression mechanical behavior and crack-merging mode of sandstone specimens containing fissure-hole combined flaws. The damage and damage characteristics of coal and rock directly affect the evolution characteristics of coal and rock seepage. Wu et al.^17^ established a permeability model to distinguish the fractured zone, plastic zone, and elastic zone around a hydraulic fracturing borehole and studied factors affecting the stress and rock permeability distribution around the borehole and the coalescence. Ding et al.^18^ studied the time-varying effect of anisotropic permeability on stress distribution, failure zone, collapse pressure, and rupture pressure in evaluating borehole wall stability. Zhang et al.^19^ presented the relationship between permeability and stress in the three-dimensional domain of fractured porous media, demonstrating that the permeability around the borehole strongly depends on the stress change induced by the borehole disturbance. The above studies have carried out in-depth research on the stress distribution theory around the hole, the failure process of the sample with holes, the effect of the stress around the hole on the fracture evolution, and the influence mechanism of fracture evolution on permeability, but there is little research on the evolution of the coal stress and the fractures during the drilling process.

In recent years, numerical simulation often has been used in the study and analysis of fracture propagation in rock materials, such as in scanning electron microscopy (SEM), atomic force microscopy (AFM),^20,21^ extended finite element method (X-FEM),^22^ boundary element method (BEM),^23,24^ and cellular automata (CA).^25^ Discontinuous medium methods, such as DEM (discrete element method),^26,27^ rock failure process analysis system (RFPA),^28,29^ and universal distinct element code (UDEC), have also been used.^30,31^ In these numerical methods, DEM is often used to simulate the mechanical behavior of some rock materials. On the basis of the principle of DEM, PFC^2D^ (particle flow code) has been greatly developed. Although PFC^2D^ is two-dimensional, it can be used to simulate many problems in mining and geotechnical engineering, such as the failure of porous rock mass, the fracture evolution mechanism, and the fracture propagation behavior in rock samples with pre-existing defects, etc.^32,33^

In this study, the formation process of boreholes has been numerically simulated using PFC^2D^. The PFC^2D^ program can better simulate the fracture propagation behavior of rigid circular particles of coal and rock materials bonded together at the contact point. First, the numerical microscopic parameters of coal seams are calibrated according to the experimental results of the coal sample. On this basis, the formation process of coal seam drilling is systematically simulated numerically, analyzing the stress variation of coal around the borehole during the formation of the coal seam under the action of stress and discussing the law of fracture evolution.

## 2 Discrete Element Model

### 2.1 Bond Models

**Figure 2:**
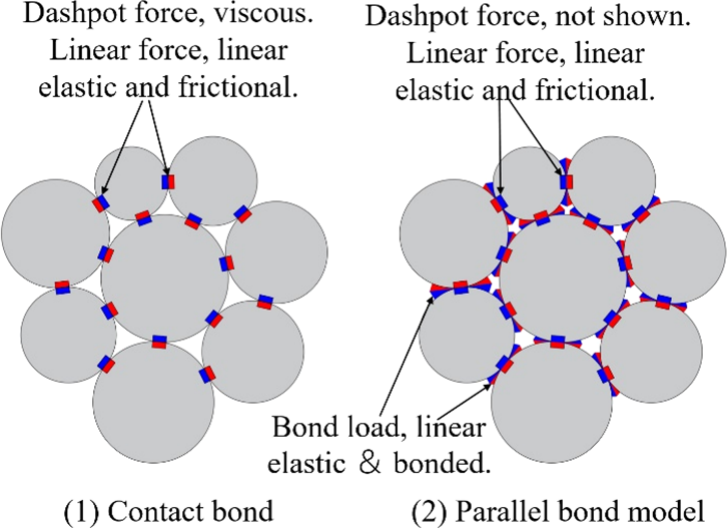
Illustration of bond models provided in PFC.

The DEM was introduced by Cundall for the analysis of rock mechanics problems. PFC^2D^ models the movement and interaction of circular particles by the DEM, as described by Cundall and Strack.^35^ In PFC, the linear-based models provide two standard bonding behaviors embodied in the contact bonds and parallel bonds, as shown in Figure 2. A contact bond can be envisioned as a pair of elastic springs (or a point of glue) with constant normal and shear stiffnesses acting at the contact point. These two springs have specified tensile and shear strengths. A parallel bond provides the mechanical behavior of a finite-sized piece of cementlike material deposited between the two contacting pieces. The parallel bond component acts in parallel with the linear component and establishes an elastic interaction between the pieces. Parallel bonds can transmit both force and moment between the pieces. Therefore, in this research, we chose the parallel bond model to carry out the numerical simulation because the parallel bond model can be more realistic for coal and rock material modeling in which the bonds may break under either tension or shearing with an associated reduction in stiffness.^33^

### 2.2 Confirmation for Simulation Parameters

**Figure 3:**
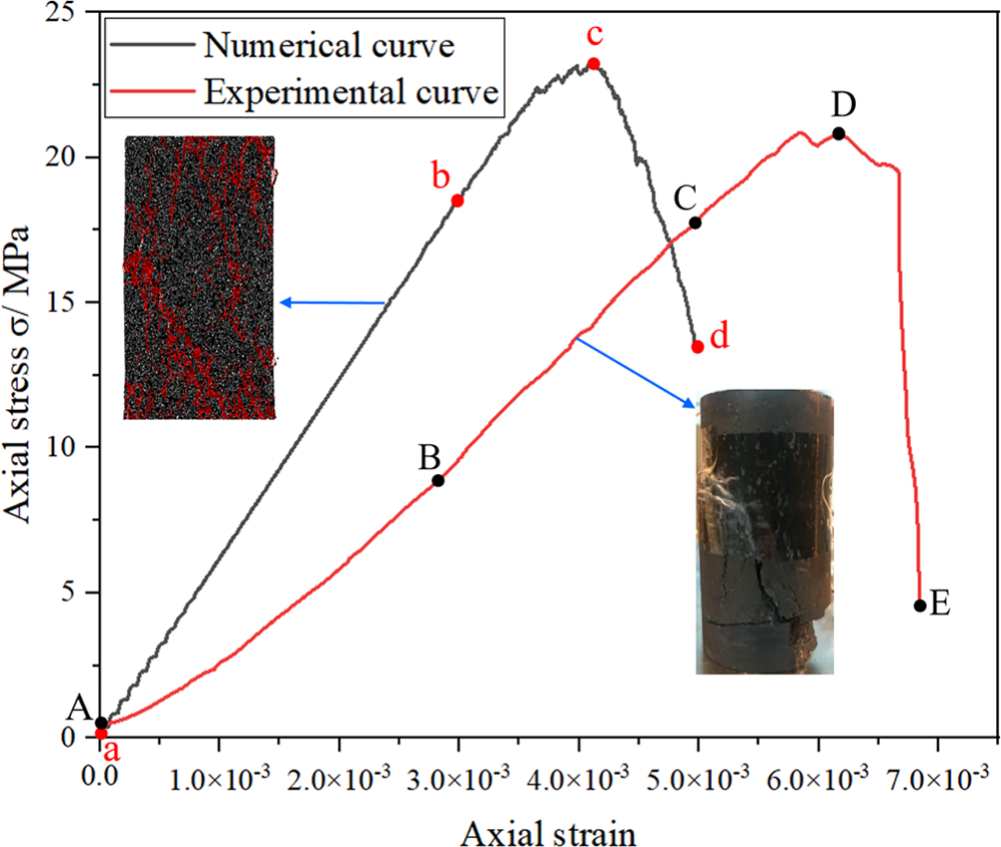
Comparison between experimental and numerical stress–strain curves of coal samples under uniaxial compression.

In order to obtain the parameters of numerical simulation, a uniaxial compression test has been carried out on the obtained coal sample, with the test coal sample being cylindrical in shape and having a a diameter of 50 mm × 100 mm. On the basis of the uniaxial compression data of coal samples obtained in the experiment, PFC^2D^ is used to simulate the failure process of uniaxial compression of coal samples and obtain the stress–strain curve. The simulated sample size is a rectangle 50 mm × 100 mm wide. Table 1 shows the parameters used in the PFC^2D^ model of the coal sample. Figure 3 shows the comparison of experimental and numerical stress–strain curves of coal samples under uniaxial compression. Figure 3 shows that the numerical simulation curve of coal samples under uniaxial compression is in good agreement with the experimental curve, including the elastic deformation stage, fracture initiation and propagation stage before peak strength, and instability failure stage after peak strength. Owing to the closure of some original fractures and pores in the test coal sample, the test specimen exhibits initial nonlinear deformation at a low stress level, which is not observed in the numerical specimen.

**Table 1:** Model Parameters of Coal Samples

parameters	values
deform emod/GPa	5.4
Kratio	1.5
particle friction coefficient, μ	0.7
Pb_deform emod/GPa	5.4
Pb_Kratio	1.5
Pb_ten/MPa	27
Pb_coh/MPa	10

### 2.3 Numerical Mode

**Figure 4:**
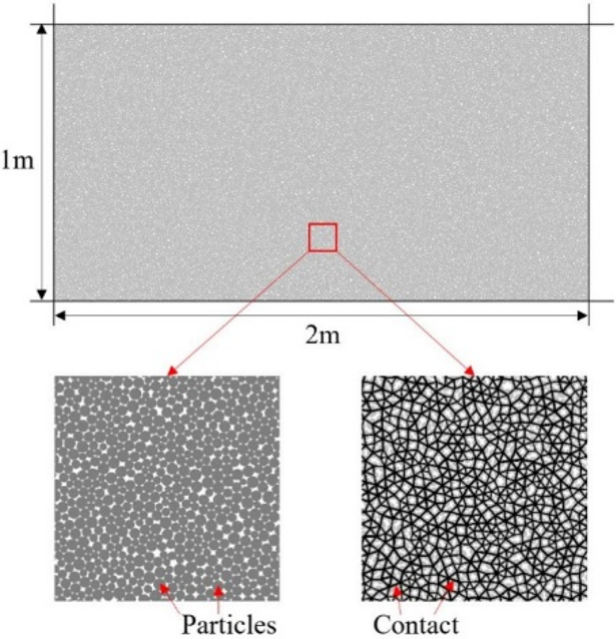
Initial numerical model of the coal seam.

Through the experimental and simulated uniaxial compression test data, the initial numerical model of the coal seam is generated by using the parameters of the model in Table 1 (Figure 4). The size of the model is 2 m × 1 m. The initial model of the coal seam is composed of 18 841 particles with a particle radius of 4–7 mm. There are 45 735 contacts between particles, and the connected porosity is 8%.

**Figure 5:**
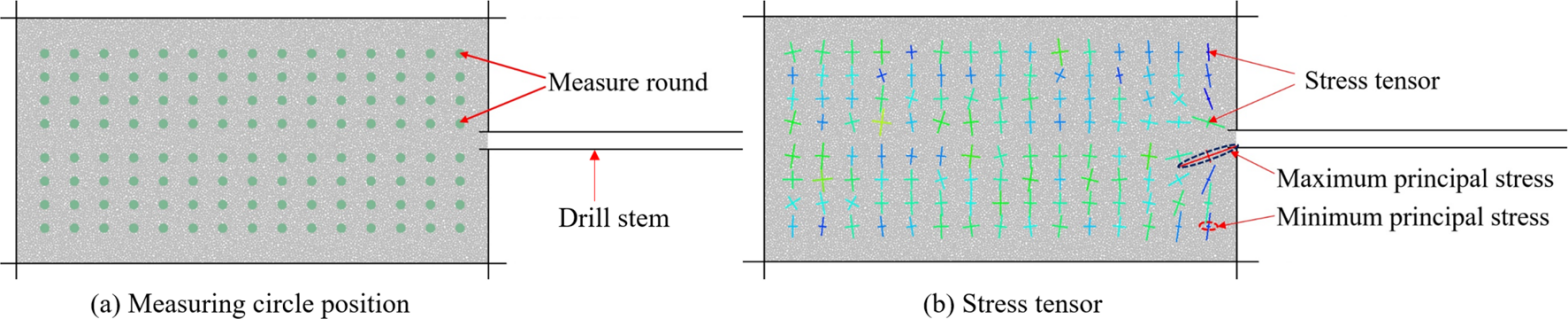
Particle model and stress tensor of the coal seam drilling process.

After the initial model of the coal seam is generated, in order to simulate the real in situ stress conditions of the underground coal seam, the vertical stress of the model is set as 5 MPa and the horizontal stress as 1 MPa, and a new drill stem is generated as a drill stem to simulate the drilling process of the drill pipe into the coal seam. The particle model of the coal seam drilling process is shown in Figure 5. The borehole radius in the model is 37.5 mm.

In the process of coal seam drilling, a measuring circle with a radius of 20 mm is arranged above and below the drilling hole, which can be used to record and monitor the stress, porosity, strain rate tensor, and component of the position of the measuring point, so as to obtain the internal stress and strain in the whole process of coal seam drilling. The layout of the measurement circle is shown in Figure 5 a. The stress tensor is generated at the center of the measuring circle, and the stress magnitude and direction of the measuring point can be seen intuitively. The stress tensor is shown in Figure 5b.

## 3 Numerical Simulation Results and Discussion

### 3.1 Coal Stress and Fracture Evolution Characteristics around the Hole

**Figure 6:**
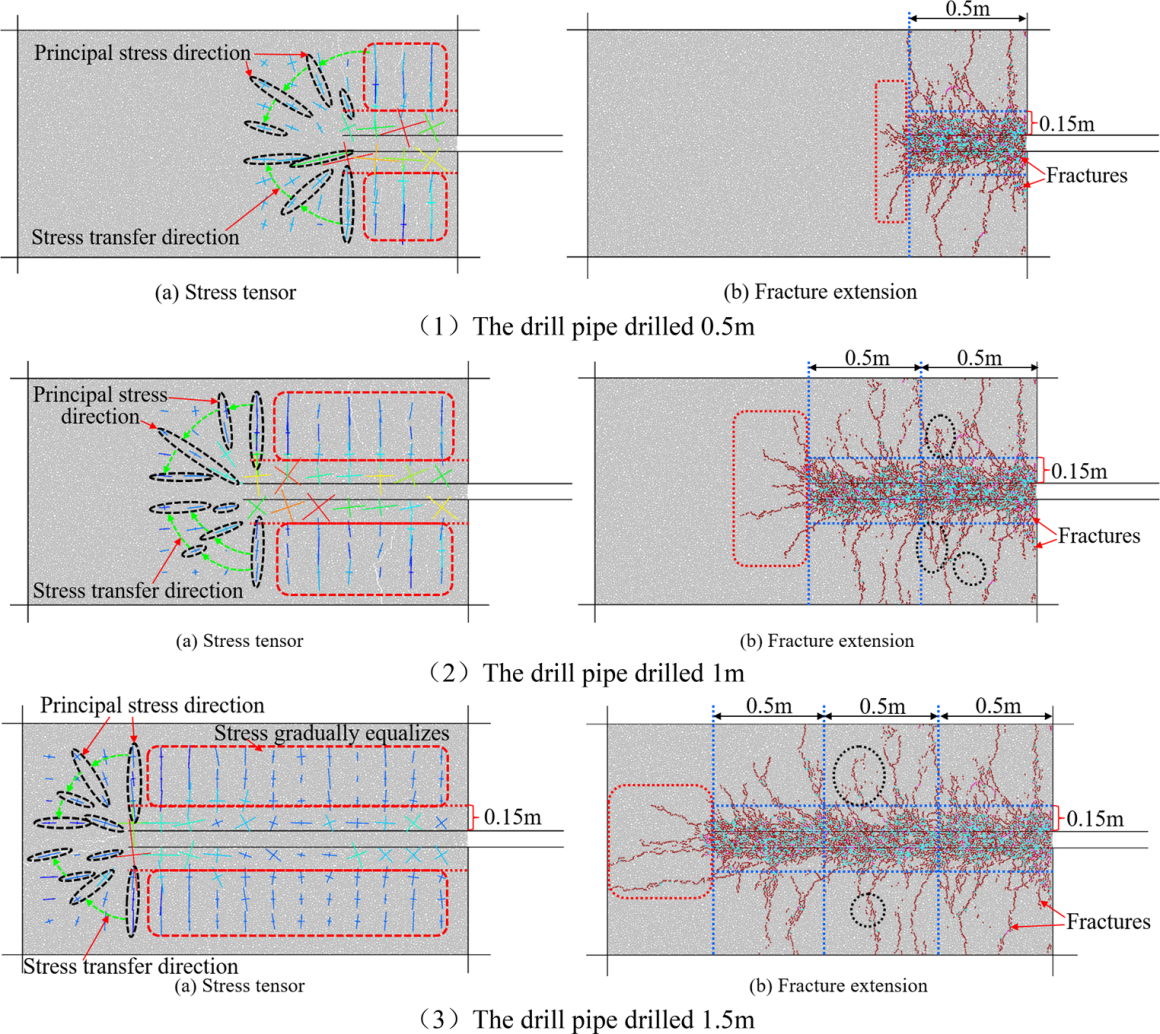
Stress and fracture division of coal around a borehole while drilling.

The drilling process is dynamic and gradual. A drilling pipe in a coal seam will cause some disturbance to the coal around the drilling hole, leading to a dynamic change of the stress state of the coal around the drilling hole. In this process, the stress change around the borehole will lead to coal damage and form a variable fracture system. Figure 6 shows the stress state of coal around the borehole and the development of corresponding fractures when the borehole is drilled at 0.5, 1, and 1.5 m.

Figure 6a shows the stress cloud map of coal in different areas around the hole during the drilling process. The cross in the figure represents the magnitude and direction of the force, the long axis of the cross represents the maximum principal stress, and the short axis of the cross represents the minimum principal stress. Figure 6b is the evolution diagram of fractures in different regions during the drilling process. It can be seen from the figure that the color of fractures is divided into three types: brown is the fracture generated by tension shear, light blue is the fracture generated by tension, and the pink color represents fractures created by pressure shearing.

Figure 6a shows the stress cloud diagram of the coal around the hole during the drilling process. The cross in the figure represents the magnitude and direction of the force, the long axis of the cross represents the maximum principal stress, and the short axis of the cross represents the minimum principal stress. Figure 6b shows the evolution of fractures during the drilling process. From Figure 6a, it can be seen that the direction of the maximum principal stress in front of the borehole gradually changes from the original vertical direction to the horizontal direction, and the minimum principal stress changes from the horizontal direction to the vertical direction during the drilling process. During the whole process of drilling, the principal stress direction within the range of 0.15 m up and down from the borehole wall near the drill bit changes from the vertical direction to the horizontal direction. When the drilling depth of the borehole is large, the magnitude and direction of the maximum principal stress and the minimum principal stress within the range of 0.15 m above and below the borehole wall behind the borehole are greatly affected by the development of fractures. During drilling, the development of coal fractures around the hole is controlled by stress, and a small amount of fractures is caused by pressure shearing. The expansion of fractures within 0.15 m from the borehole is mainly due to the tension and tensile shear of the coal body, while the tensile shearing action greater than 0.15 m is the main reason for the development of fractures. From Figure 6b, it can be seen that when the borehole is drilled 0.5 m, there are fewer fractures in front of the borehole, and the fractures on both sides of the borehole wall are more obvious; when the drilling depth is increased to 1 m, the development of fractures in front of the borehole is more obvious than that of 0.5 m. The development direction of the fractures is tree root-like, and the distribution is relatively uniform. When the borehole is drilled for 1.5 m, the development direction of the fracture in front of the borehole tends to be consistent with the drilling direction, and the length of the fracture expansion increases significantly.

Along the axial direction of the borehole, the coal fractures around the hole are divided into two areas: the front end and the back end. The rear end zone of the fracture is divided every 0.5 m with different drilling depths. The three zones I, II, and III are divided in part (b) in Figure 6. From the evolution characteristics of the fractures in the fracture area at the front end of the borehole, it can be seen that there are fewer fissures developed at the front end of the borehole when the borehole is drilled 0.5 m, and the fissures develop at a shallow depth; when the drilling depth increases to 1 m, the development of cracks at the front end of the drilling hole is more obvious, the distribution of the development direction of the cracks is more uniform and divergent, and the development depth of the cracks increases. When the borehole is drilled for 1.5 m, the front end of the borehole develops mainly tensile fractures along the drilling direction. By observing part (b) in parts (1) and (2) of Figure 6, it can be seen that in the process of a drilling depth of 0.5–1 m, the coal within a distance of 0.15 m from the hole wall in the rear fracture zone I is strongly disturbed by the drilling, and the development degree of fractures is dense. However, when the distance from the hole wall is greater than 0.15 m, it gradually develops on the basis of the original fractures. It can be seen intuitively that the number of fractures increases in the circle area of the black dotted line in part (b) in Figure 6, part (2). By comparing the vertical fracture zone on both sides of the borehole in parts (2) and (3) of Figure 6 with part (b), it can be seen that the fracture evolution characteristics in the rear fracture zone II during the drilling depth of 1–1.5 m are the same as those during the drilling depth of 0.5– 1 m.

**Figure 7:**
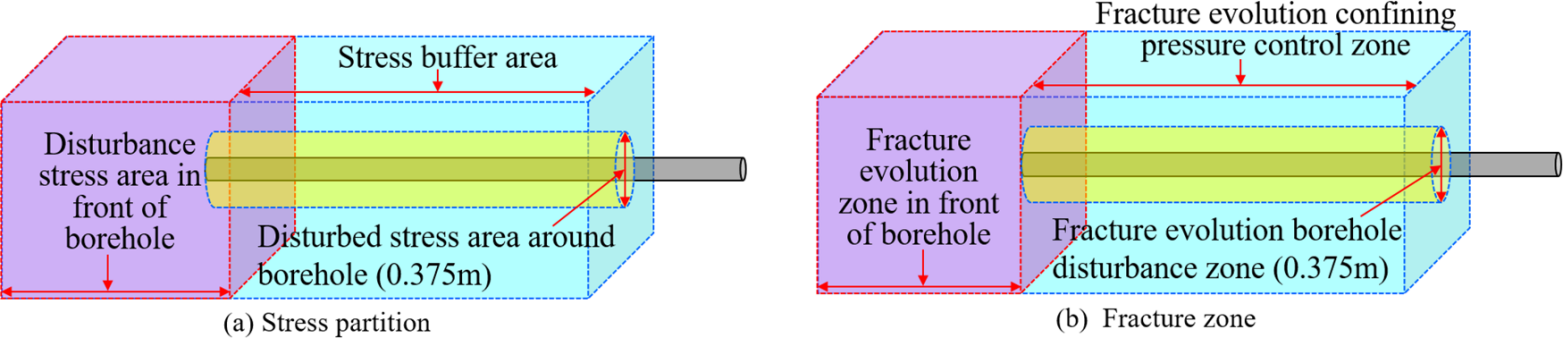
Stress zoning and fracture zoning.

It can be seen from the analysis in Figure 6a that the stress of the drilled coal has the highest stress concentration in front of the borehole, and the coal is also most seriously disturbed during the hole-forming process, so the stress disturbance zone at the front end of the borehole is divided. It can be seen as the purple area in Figure 7a. When the peak strength of coal within 0.15 m from the borehole is smaller than the concentrated stress, the fractures gradually develop, and the evolution of the fractures releases the concentrated stress on the coal, thus dividing the disturbed stress area around the borehole, as shown in the yellow area in Figure 7a. As the drilling depth of the borehole increases, the concentrated stress zone gradually moves toward the direction of the borehole, and the stress of the coal behind the borehole tends to be in a balanced state until the coal around the borehole reaches a new equilibrium and stable state. The stress buffer zone is thus divided, as shown in the blue area in Figure 7a.

During the drilling process, the stress of the coal around the hole changes dynamically, and the fractures in the coal gradually expand or close with the change of the stress. Through an analysis of Figure 6b, it can be seen that the fracture evolution zone at the front of the borehole is divided according to the evolution characteristics of the fractures in front of the borehole during the drilling process, as shown in the purple area in Figure 7b. When the peak strength of the coal within 0.15 m from the borehole is less than the magnitude of the concentrated stress, a large number of fractures develop, thus dividing the borehole disturbance zone of the fracture evolution, as shown in the yellow area in Figure 7a. Controlled by the confining pressure, fractures gradually expand to the deep coal in the range greater than 0.15 m away from the borehole, thus dividing the confining pressure control zone of the fracture evolution, as shown in the blue area in Figure 7a.

### 3.2 Relationship between Stress and Fracture Evolution of Coal around a Hole

**Figure 8:**
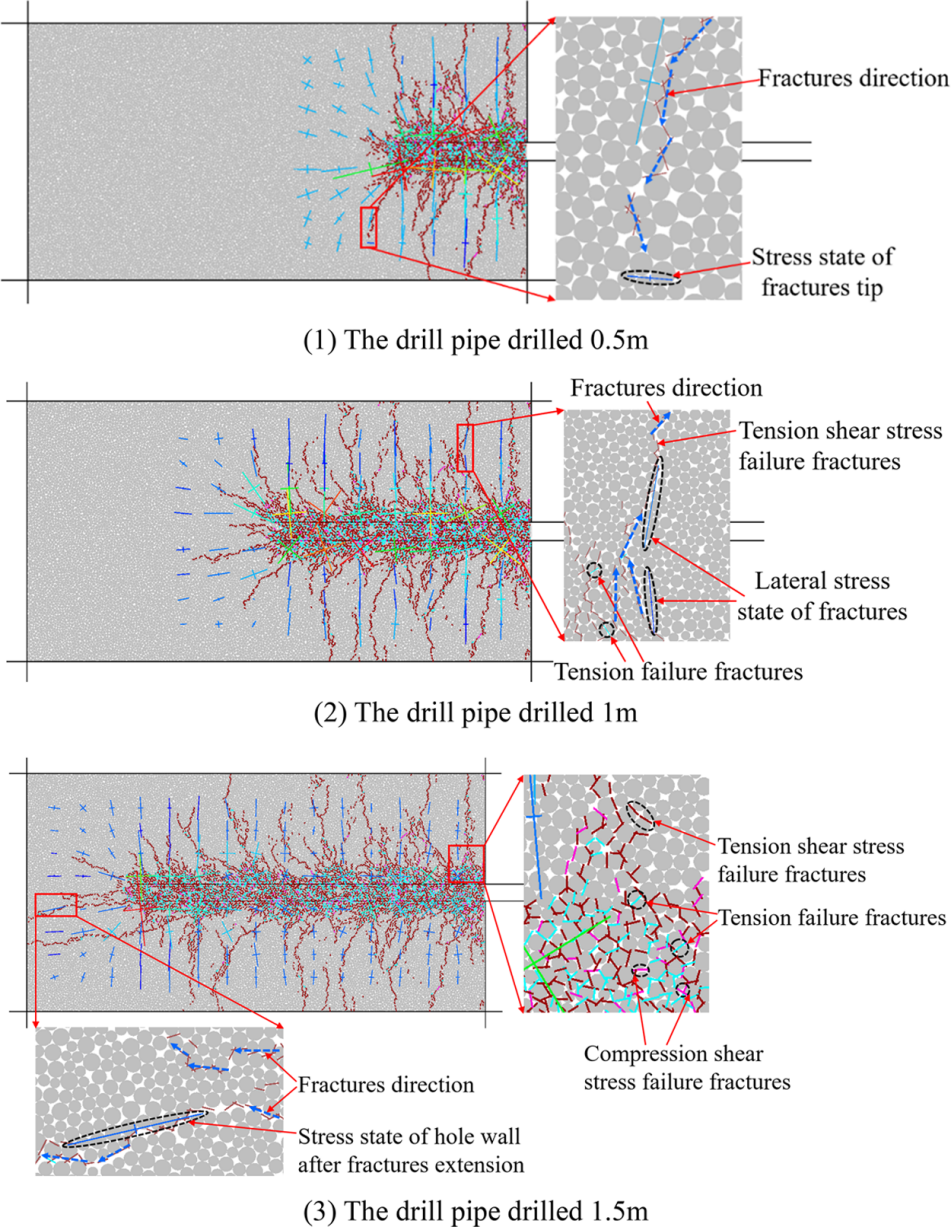
Stress and fracture evolution pictures of coal around a borehole.

In order to explain the evolution relationship of stress and fracture of coal around a borehole in coal seams, the evolution diagram of stress and the corresponding fracture of coal around the borehole in the coal seam shown in Figure 8 is obtained by combining the evolution picture of stress and fracture in Figure 7.

It can be seen from part (1) in Figure 8 that when the borehole is drilled for 0.5 m, the evolution of internal cracks is observed. When the coal is sheared under the action of the maximum principal stress, the maximum principal stress at the tip of the crack will be released and rapidly decrease. It can be seen from Figure 8, part (2) that when the vertical stress is the maximum principal stress, the tensile shear action of the coal around the pore is the main reason for the development of the fracture. The fracture expansion will change the direction of the maximum principal stress of the coal on the side of the fracture, and the direction of the fracture expansion will gradually move toward the direction of the maximum principal stress. Figure 8, part (3) shows that when the maximum principal stress of coal at the front of borehole is horizontal shear stress during drilling, the direction of the maximum principal stress received by the fracture hole wall also shifts along with the evolution direction of the fissure. The internal stress of the coal drilled into the borehole has the highest stress concentration at the front end of the borehole, and the coal is also most seriously disturbed during the pore-forming process. When the peak strength of the coal mass is less than the magnitude of the concentrated stress, the fractures gradually develop, and the evolution of the fractures releases the concentrated stress on the coal mass. As the drilling depth of the borehole increases, the concentrated stress zone gradually moves toward the direction of the borehole, and the stress of the coal mass at the rear end of the borehole tends to a balanced state until the coal mass around the borehole reaches a new equilibrium and stable state.

### 3.3 Variation Process of the Stress Field of Coal around a Hole

**Figure 9:**
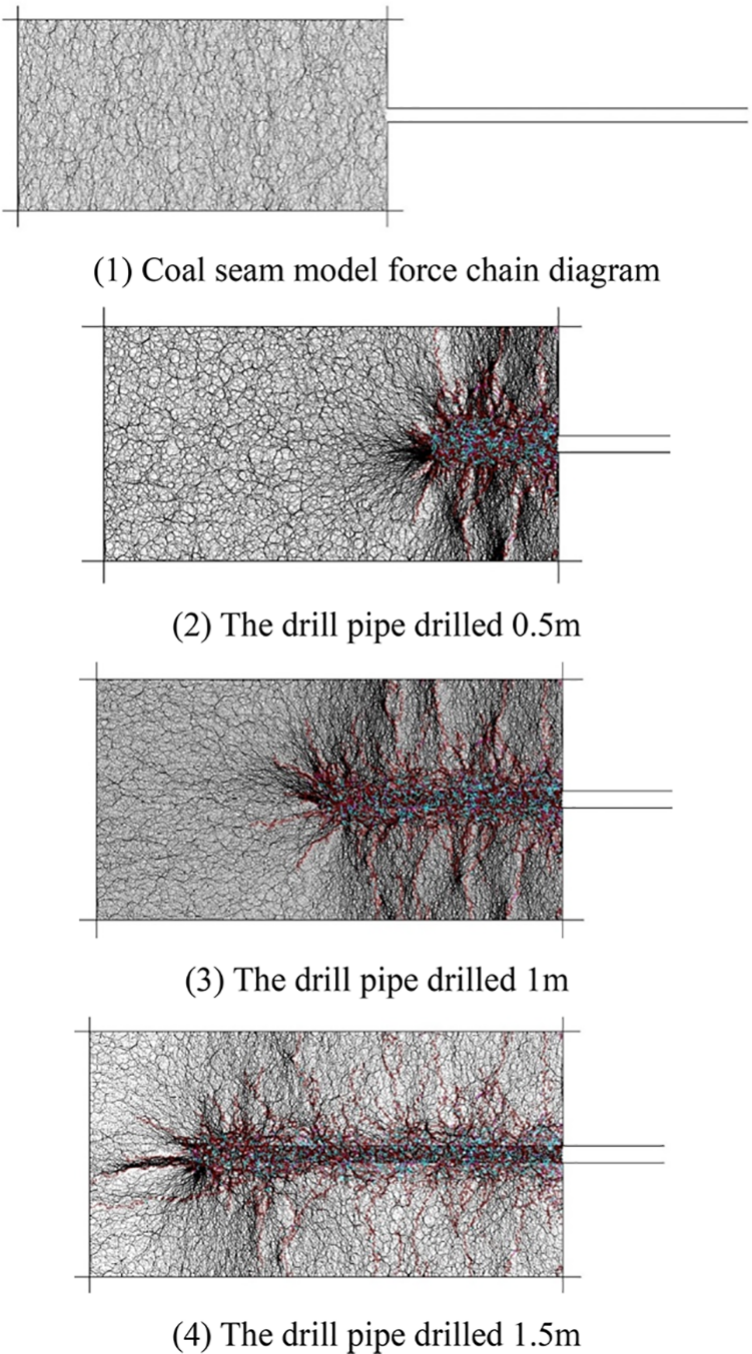
Evolution diagram of the contact force chain and fracture around a borehole in a coal seam.

Figure 9 shows the parallel bond force diagram inside the coal seam during the drilling process of the coal seam. The parallel bonding forces in the figure are represented by discrete straight line segments. The thickness and direction of the black line correspond to the magnitude and direction of the force, respectively.

It can be seen from Figure 9 , part (1) that before the borehole is drilled, the parallel cohesive force inside the coal seam is evenly distributed. When the drilling depth of the borehole is 0.5 m, it can be seen from the distribution of the parallel bonding force shown in Figure 9 , part (2) that the concentrated stress in front of the borehole is relatively large, and there is also local stress concentration around the borehole. At this time, the concentration of black lines is large, the parallel cohesion force increases, and coal fractures are in the initial stage of development. When the drilling depth of the borehole increases to 1 m, there is also a stress concentration phenomenon in the coal in front of the borehole. At this time, the concentration of black lines decreases, and the parallel bonding force decreases accordingly. Coal fractures in the stress concentration area gradually develop, while the rear coal fractures gradually develop and the local stress concentration decreases. When the borehole is drilled to 1.5 m, there is still a stress concentration area in front of the borehole, and the fractures expand to the deep coal seam obviously, while the continuous development of coal fractures around the back hole leads to the release of coal stress. At this time, the concentration of black lines is greatly reduced, the parallel bonding force is reduced, and the stress tends to be in an equilibrium state.

### 3.4 Distribution Characteristics of Cracks and Contact Forces

**Figure 10:**
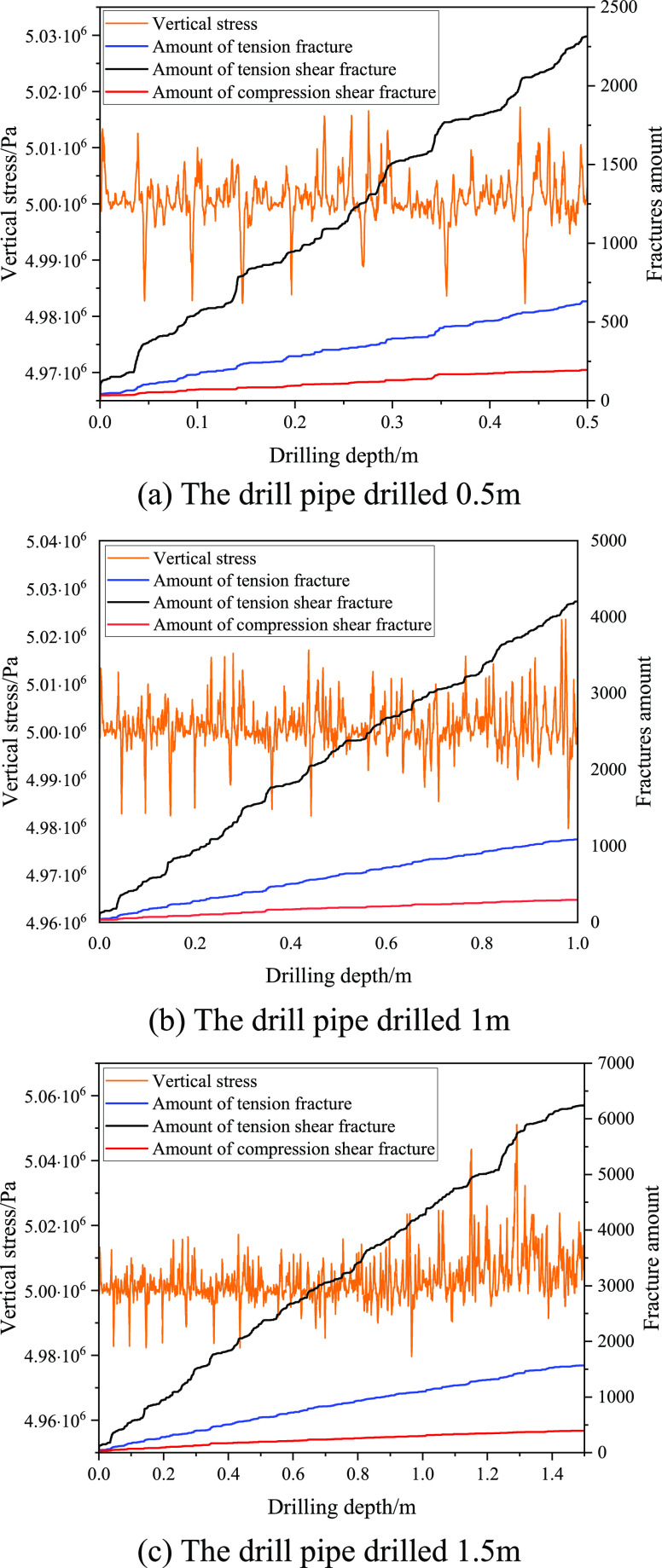
Stress state and fracture amount of coal around a borehole while drilling.

In order to obtain the change of stress state and fracture number of coal around a borehole during drilling, a measurement circle has been used to obtain the stress change of coal above the hole wall to study the change of stress state and fracture number of coal around the borehole during drilling. The number of fractures and stress changes caused by borehole disturbance in coal when drilling 0.5, 1, and 1.5 m are statistically analyzed, shown in Figure 10a, b, and c, respectively.

It can be seen from Figure 10 that with drilling, the interaction between the drill pipe and coal makes the coal stress around the hole fluctuate around the original vertical stress of the coal seam, and the state of coal in front of drilling is stress concentration–damage–stress release–stress concentration. With the increase of drilling depth, the stress of coal behind the drilling gradually tends to an equilibrium state, and the number of fractures will increase greatly.

**Figure 11:**
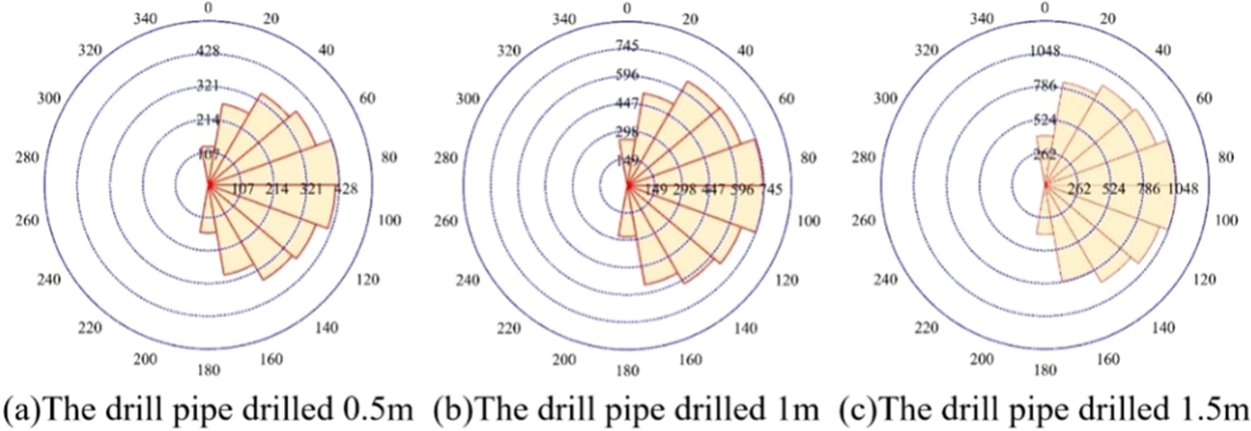
Fracture rose diagram of coal around a borehole in a coal seam while drilling.

A rose diagram is a statistical diagram resembling a rose, indicating the trend or tendency and number of fractures. It reflects the development degree of fractures in each group within the observation range, and one can clearly see the dominant orientation. Figure 11 is the rose diagram of the fracture evolution of coal around different depths drilled by coal seam drilling. As can be seen from Figure 11, when drilling 0.5, 1, and 1.5 m, fractures in the model gradually developed under the influence of the drilling disturbance. The direction with an angle from 70° to 110° horizontal to the drilling hole is the main direction of the fracture development, and the number of fractures accounts for the largest proportion, followed by those in the direction of 30°–70° and 110°–150°. The number of fractures increases with the increase of the drilling depth.

**Figure 12:**
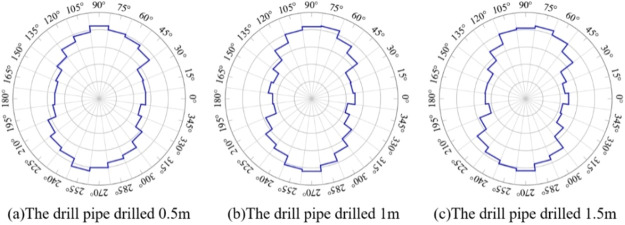
Contact normal force distribution around a borehole in a coal seam while drilling.

An important means to study mesoscopic media is analyzing the action direction of normal and tangential contact forces between the particle system and studying the law of force transfer. Figure 12 is the polar axis diagram of the particle contact normal force distribution at different depths of coal seam drilling. The solid blue line in the figure represents the statistical results of the contact normal force distribution of particles in the numerical model. It can be seen from Figure 12 that when drilling 0.5, 1, and 1.5 m, the rose diagram of the contact normal force of particles in the model presents an oval shape and the coal seam model presents anisotropy under the influence of the drilling disturbance, which is consistent with previous research results on the contact normal direction.^34^ With the increase of the drilling depth, the pressure relief of coal during drilling will lead to the decrease of the vertical normal force. In the process of pipe drilling, the vertical stress of coal in the front end of the drilling pipe gradually turns to horizontal stress, leading to the decrease of the horizontal normal force inside the model, which is consistent with the stress state of coal around the hole during the drilling process of a coal seam as shown in Figure 6.

**Figure 13:**
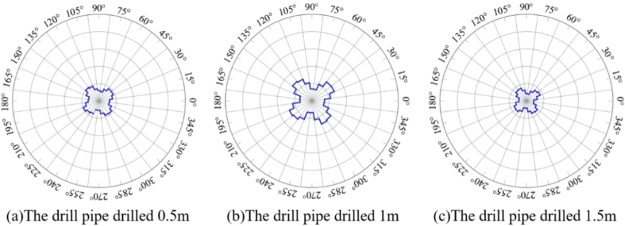
Distribution of the contact tangential force around a borehole in a coal seam while drilling.

Figure 13 is the polar axis diagram of the particle contact tangential force distribution in coal seam drilling at different depths. The solid blue line in the figure represents the statistical results of the tangential force distribution in contact with particles in the numerical model. Figure 13 shows that the rose diagram of the contact tangential force inside the model is petal shaped, and the coal seam model presents anisotropy, which is consistent with previous research results on contact tangential force.^34^ By comparing parts (a) and (b) in Figure 13, it can be seen that when the drilling depth is less than half of the total length, the tangential force inside the model shows an increasing trend with the increase of drilling depth. According to parts (b) and (c), when the drilling depth exceeds half of the total length, the tangential force inside the model decreases with the increase of the drilling depth. This is because the increase of the drilling depth will lead to a gradual decrease in the shear stress at the back end of the drill hole, while the stress concentration at the front end of the drill hole will lead to an increase in the shear stress. The results are consistent with the analysis results of the stress state of coal around the rear end of coal seam drilling shown in Figure 6 , parts (2) and (3).

## 4 Conclusions

In order to study the influence law of stress and fracture evolution of coal around a borehole during coal seam drilling in an underground coal mine, the particle flow program PFC^2D^ is used to analyze the stress and fracture evolution characteristics of coal around a borehole during coal seam drilling. According to the characteristics of the coal seam stress field and its influence on the development of coal fractures around the hole, it can provide theoretical support for the efficient extraction of coal seam gas. Through the simulated results, the following conclusions can be summarized:

(1) During the drilling process of the coal seam drilling, the maximum principal stress in front of the drilling hole gradually changes from the original vertical stress to a horizontal stress, the development of coal fractures around the hole is controlled by the stress, and a small amount of fractures is caused by the pressure-shearing effect. The expansion of fractures within 0.15 m from the borehole is mainly caused by the tension and tensile shear of the coal, and a tension-shearing action greater than 0.15 m is the main reason for the development of fractures.
(2) The evolution relationship of coal stress and fracture around the coal seam borehole is explained. The crack propagation will change the direction of the maximum principal stress of the coal on the side of the fracture, and the direction of the fracture propagation will gradually tend to the direction of the maximum principal stress; during the drilling process, when the maximum principal stress of the coal in front of the drill pipe is the horizontal stress, the stress on the inner hole wall of the fracture generated by the horizontal shear stress will also transfer with the evolution direction of the fracture.
(3) By analysis of the direction and quantity of coal fracture evolution during the drilling process of a coal seam, the development degree of fractures is indicated. When the borehole was drilled for 0.5, 1, and 1.5 m, the fractures in the model gradually developed, and the direction with an included angle of 70°–110° with the borehole level was the dominant orientation for the development of fractures, and the number of fractures accounted for the largest proportion.
(4) With the increase of the drilling depth, the pressure relief of the coal during the drilling process and the gradual direction change of the maximum principal stress of the coal in front of the drill pipe from the vertical stress to the horizontal direction are the reasons for the decrease of the normal force in the vertical direction. The stress concentration at the front of the borehole will lead to the increase of the shear stress and the gradual decrease of the shear stress at the back of the borehole, which manifests as the tangential stress first increasing and then decreasing .
